# Addressing safety risks in integrated care programs for older people living at home: a scoping review

**DOI:** 10.1186/s12877-020-1482-7

**Published:** 2020-02-28

**Authors:** Manon Lette, Eliva A. Ambugo, Terje P. Hagen, Giel Nijpels, Caroline A. Baan, Simone R. de Bruin

**Affiliations:** 1 Amsterdam Public Health research institute, Department of General Practice and Elderly Care Medicine, Amsterdam UMC – VU University Amsterdam, Amsterdam, the Netherlands; 2 Centre for Nutrition, Prevention and Health Services research, National Institute for Public Health and the Environment, Bilthoven, the Netherlands; 3 Department of Health Management and Health Economics, Institute of Health and Society, University of Oslo, Oslo, Norway; 4 Scientific Center for Transformation in Care and Welfare (Tranzo), University of Tilburg, Tilburg, the Netherlands

**Keywords:** Integrated care, Safety, Risks, Prevention, Elderly, Older people living at home, Primary care, Community care, Scoping review

## Abstract

**Background:**

Many older people live at home, often with complex and chronic health and social care needs. Integrated care programs are increasingly being implemented as a way to better address these needs. To support older people living at home, it is also essential to maintain their safety. Integrated care programs have the potential to address a wide range of risks and problems that could undermine older people’s ability to live independently at home. The aim of this scoping review is to provide insight into how integrated care programs address safety risks faced by older people living at home - an area that is rather underexplored.

**Methods:**

Safety was conceptualised as preventing or reducing the risk of problems, associated with individual functioning and behaviour, social and physical environments, and health and social care management, which could undermine older people’s ability to live independently at home. For this scoping review a systematic literature search was performed to identify papers describing integrated care programs where at least one intervention component addressed safety risks. Data were extracted on the programs’ characteristics, safety risks addressed, and the activities and interventions used to address them.

**Results:**

None of the 11 programs included in this review explicitly mentioned safety in their goals. Nevertheless, following the principles of our conceptual framework, the programs appeared to address risks in multiple domains. Most attention was paid to risks related to older people’s functioning, behaviour, and the health and social care they receive. Risks related to people’s physical and social environments received less attention.

**Conclusion:**

Even though prevention of safety risks is not an explicit goal of integrated care programs, the programs address a wide range of risks on multiple domains. The need to address social and environmental risks is becoming increasingly important given the growing number of people receiving care and support at home. Prioritising a multidimensional approach to safety in integrated care programs could enhance the ability of health and social care systems to support older people to live safely at home.

## Introduction

All over the world, countries are facing aging populations. Old age is associated with an increased prevalence of chronic conditions, functional decline and frailty, which often results in chronic and complex health and social care needs [1–3]. As a result, the demand for health and social care services is increasing [4]. In response to the growing pressures on the health and social care systems, older people are being stimulated to live independently at home for as long as possible. The majority of older people do indeed ‘age in place’ [5–7], often with support from a range of formal and informal care providers.

In an effort to better address older people’s chronic health and social care needs, integrated care programs for older people living at home are increasingly being implemented in primary and community care settings [8–11]. Although different definitions and models of integrated care exist [12–14], integrated care is generally characterised as a proactive and person-centred approach to care and support that is seamlessly coordinated across multiple professional disciplines and care interfaces, and responsive to the risks and needs older people may face on various domains of life [10, 11]. Integrated care programs come in many shapes and forms, and can pursue different aims ranging from improving functional outcomes, quality of life, quality of care and efficiency [15]. The organisation of integrated primary and community care in considered an essential step towards supporting older people to live independently at home for as long as possible [16].

In order for older people to successfully age in place, it is also important to maintain their safety. Research on patient safety has traditionally focused on the “prevention of errors and adverse effects associated with health care” ( [17], par. 1). However, older people encounter limitations in multiple domains of life, which could also pose risks to their ability to live safely and independently at home [18–25]. This implies a need to look at older people’s safety at home from a broader perspective. Lau et al. (2007) [23] proposed a model for health-related safety that extends beyond the institutional settings and includes a wide range of risk factors in multiple domains of life. Following the principles of this model, risks relate to older people’s health and functioning (e.g., physical or cognitive decline), their lifestyle and behaviour (e.g., dietary intake, self-care, medication adherence), their social or physical environments (e.g., social isolation, caregiver burden, hazards in the home) and the health and social care they receive (e.g., medication errors, communication failures, fragmentation of care) [18–27]. This wide range of risks could lead to a multitude of problems that challenges people’s ability to live safely at home, and could ultimately result in emergency department visits, (re) hospitalisation, institutionalisation and mortality [23]. It is therefore deemed important to address such risks and focus efforts on preventing problems.

Given its proactive, interdisciplinary and comprehensive character, integrated care may provide opportunities to address this range of risks to older people’s safety at home [28]. However, the extent to which integrated care programs currently do this is unclear. There is limited evidence for the effectiveness of preventative integrated care for older people [8, 29], and integrated care programs throughout Europe have been shown to lack a focus on preventative aspects [30]. This suggests a need to increase our awareness of types of risks that are currently considered in integrated care programs, and the scope of activities and interventions that are employed to tackle these risks. Such insights will help researchers and policy makers to identify knowledge gaps, and promote their understanding of what might additionally be needed to properly support older people to live safely at home [31, 32].

To address this knowledge gap in the literature, this study aims to provide insight into how integrated care programs currently address safety risks for older people living at home. This will be done by reviewing integrated care programs published in the scientific literature, which may have various approaches and aims. We will use the principles of Lau et al.’s broad perspective on safety to examine how these programs address safety risks on multiple domains of people’s lives. This review will answer two questions: 1) which safety risks are addressed in integrated care programs for older people living at home, and 2) which activities and interventions are used to address these risks?

## Methods

The research questions in this study can be addressed appropriately by carrying out a scoping review [33]. As such, this paper follows the Preferred Reporting Items for Systematic Reviews and Meta-Analyses (PRISMA) guidelines for scoping reviews [34].

### Search strategy

We conducted a systematic literature search in the Embase and Medline electronic databases with support from a librarian. We searched for papers that described integrated care programs for older people living at home, and that included intervention components that addressed safety. We limited our search to papers published in English, Dutch and Norwegian given our language abilities as authors; and searched for papers published between January 2007 and February 2018. The search combined terms to identify: 1) the target group (e.g., *older people, elderly people, frail, aging, geriatric*), 2) the care setting (e.g., *living in the community, living at home, community-dwelling, home dwelling, independent living, home care, home nursing*), 3) integrated care programs (e.g., *integrated care, care coordination, case management, comprehensive care, managed care, interdisciplinary care, person-centred care*), and 4) safety (e.g., *home care safety, patient safety, preventable harm, risk reduction, prevention of adverse events/death/hospitalisations, prevention of risk/medication error/malnutrition/deterioration/social isolation*). The detailed search terms and steps are available in Additional file 1. In addition to searching the electronic databases, we identified other relevant papers through reference tracking and manual searches.

### Inclusion criteria and definitions

Studies that met the following criteria were eligible for inclusion:

1. *Population*: the program in the study targets older people (≥65 years of age) living at home with multiple health and social care needs.
2. *Setting*: the program in the study is delivered in older peoples’ homes or in the primary or community care setting.
3. *Integrated care*: the program in the study is an integrated care program. For the purpose of this review, integrated care programs were defined as programs that complied with the following three principles of integrated care drawn from the Chronic Care Model and related models [12, 35–38]: 1) interdisciplinarity, meaning that the program aimed to involve professionals from at least two different health and social care professions, 2) comprehensiveness, that is, the program aimed to focus on participants needs in multiple domains of life (i.e., physical, cognitive, psychological, social and environmental), and 3) person-centeredness, meaning that the program aimed to centre care around older people’s needs and wishes, and/or aimed to actively involve older people in the care process.
4. *Design*: the study addressed the evaluation of an integrated care program, meaning that we included studies that published program protocols and descriptions, as well as process and outcome evaluations of programs.
5. *Safety*: the program in the study contained explicit intervention components that address older people’s safety at home. For this review, safety was conceptualised as preventing or reducing the risk of problems that could undermine older people’s ability to live independently at home. Following the principles of Lau et al.’s framework for health-related safety [23], risks could be associated with individual functioning and behaviour (e.g., physical or cognitive decline, medication adherence, dietary intake), social and physical environments (e.g., caregiver burden, social isolation, in-home hazards), and health and social care management (e.g., polypharmacy, care transitions, over- or under treatment) [18, 23].

Studies were excluded when: 1) they described programs addressing populations with specific diseases (e.g., patients with diabetes or chronic obstructive pulmonary disorder), 2) they described programs delivered in the hospital or nursing home, or 3) they were not published in peer-reviewed scientific journals.

### Study selection

After performing the literature search, two reviewers (ML and EA) independently screened the titles and abstracts of the identified papers to assess their relevance based on the first four criteria. Full text papers were retrieved for the studies that both reviewers considered relevant. The reviewers then assessed the studies for eligibility based on their full text using all five inclusion criteria. Any disagreements between the reviewers were resolved by consulting a third reviewer (SdB) and then reaching a consensus.

### Data extraction and analyses

**Fig. 1:**
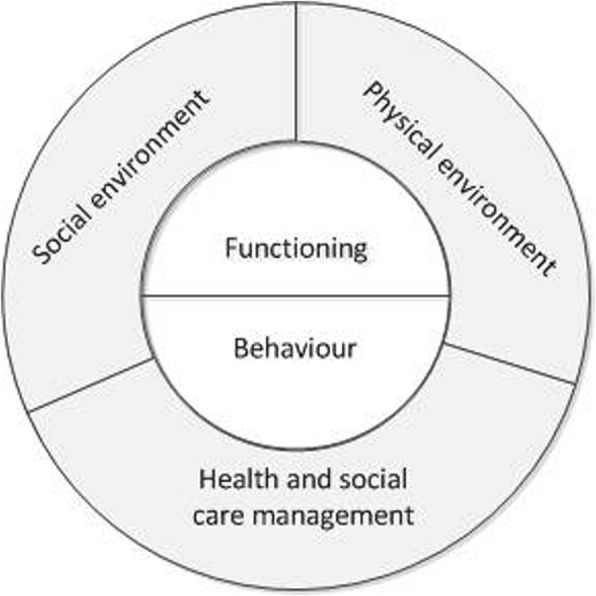
Domains of safety risks (based on the principles of Lau et al.’s framework for health-related safety and additional literature [18–24]). This figure presents the five domains that were used to categorise the safety risks and interventions identified in the included studies

Two reviewers (ML and EA) independently extracted relevant data from the included studies using a predefined template. Data were extracted on the general characteristics of the integrated care program (i.e., country, setting and target group, goals, and how programs address the elements of integrated care), and on the safety-related intervention components of the program (i.e., the risks addressed, and the interventions and activities used to address them). These safety-related components were categorised into five domains of safety risks, which were defined based on the principles of Lau et al.’s framework for health-related safety and additional literature [18–24]. The domains included client functioning, client behaviour, social environment, physical environment, and health and social care management (see Fig. 1; also see inclusion criteria 5). This conceptualisation of safety was used as a ‘new’ lens to look at existing information. Therefore, the extracted safety-related data consisted of both information that was labelled as safety-related in the included studies, as well as information that was interpreted as safety-related by the two reviewers, based on this conceptualisation. Data on the activities and interventions used to address risks were limited to the descriptions and reasoning provided in the included studies. An examination of intervention mechanisms of the identified activities and interventions was beyond the scope of this review. Program components not related to any of the five safety domains were not reported.

## Results

**Fig. 2:**
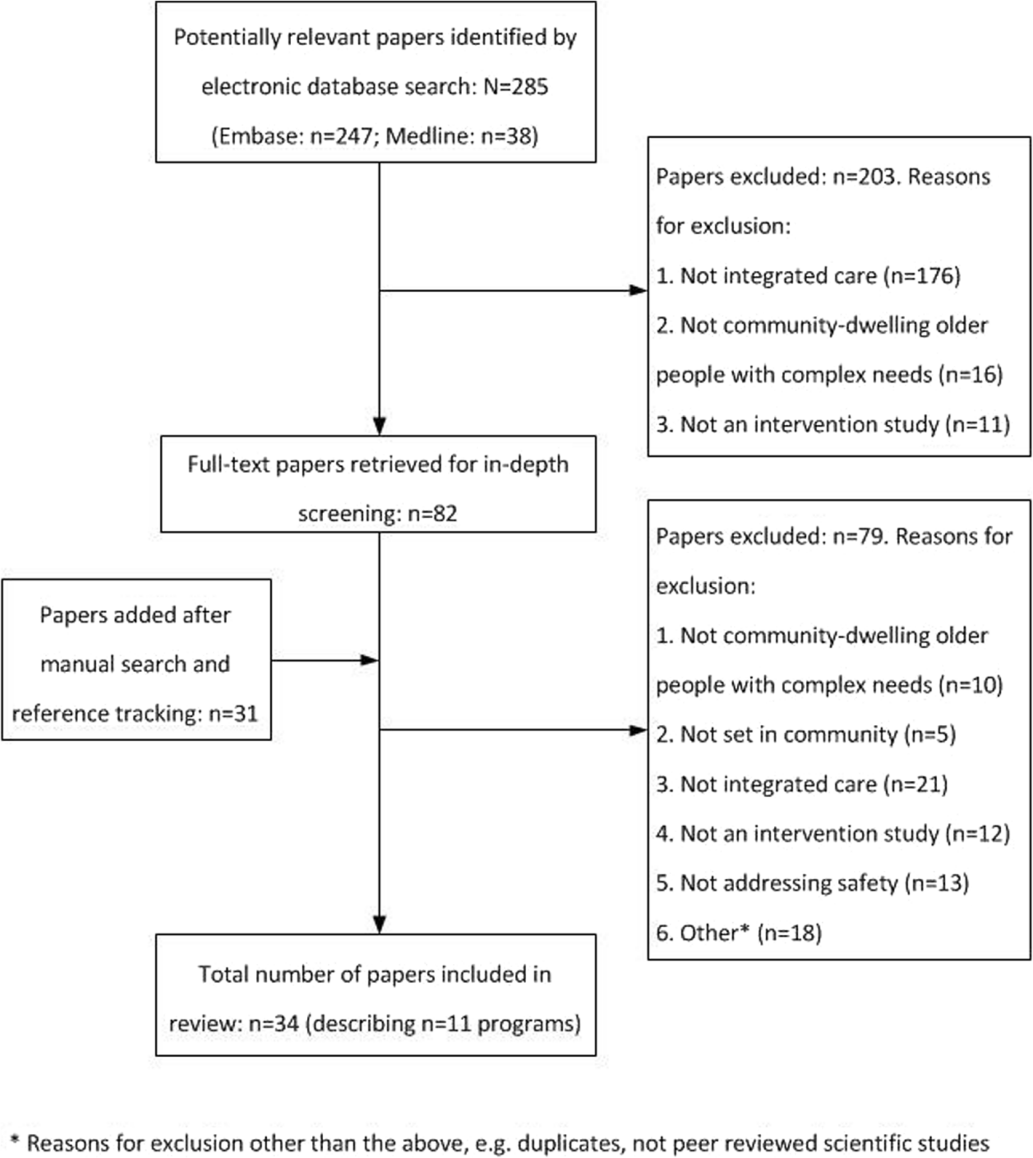
Flowchart of study selection process. This figure provides an overview of the different steps taken during the study selection process

A flowchart of the study selection process is presented in Fig. 2. A total of 285 papers were identified through the systematic search of the electronic databases. After their titles and abstracts were screened, 82 publications were selected for full text screening. An additional 31 papers were selected for full-text screening after reference tracking. Full-text screening resulted in a total of eleven integrated care programs, described across 34 publications, that were included in this review.

### Characteristics of included programs

The eleven programs included in this review are presented in Tables 1-3. As shown in Table 1, the programs were from the United States (*n* = 4), the Netherlands (n = 4), Sweden (*n* = 2) and Switzerland (*n* = 1). Consistent with our inclusion criteria, included programs targeted people with complex health and social care needs. Some programs explicitly stated that they screened for participants who were cognitively unimpaired (*n* = 6 programs), or who were at risk of (or experiencing) functional limitations (*n* = 5). None of the included programs explicitly mentioned safety or improvements in safety as the program’s goal. Generally, programs could be categorised into those that aimed to: 1) prevent or delay functional decline or improve health outcomes (*n* = 9); 2) reduce service use or costs (*n* = 6); 3) improve quality of life (*n* = 4); and 4) improve quality of care (*n* = 2).

**Table 1:** Characteristics of included integrated care programs (abbreviation definitions below)

Country	Program name	Setting + target group	Goal of program	Elements of integrated care addressed in program		
				*Interdisciplinary*	*Comprehensive*	*Person-centred*
United States	ABLE [39, 40]	People aged ≥70 years from an urban community who were cognitively unimpaired and reported some functional difficulties.	Reduce functional difficulties by modifying behavioural and environmental contributors to functional decline.	OT and PT performed home visits and telephone consultations, and together coordinated their activities.	Program targeted participants’ physical functioning, behaviour and physical environment.	Intervention focused on problems identified and prioritised by older people.
United States	CAPABLE [41–43]	Low-income people aged ≥65 years from an urban community who were cognitively unimpaired, and who reported some functional difficulties.	Reduce the impact of disability among low income adults by addressing individual abilities and the home environment.	OT and RN performed home visits, and licensed handyman performed home modifications.	Program targeted participants’ physical health and functioning, psychological health, behaviour and physical environment.	Older people were involved in identifying and prioritising problem areas, and developing action plans.
United States	Guided Care [44–47]	Older people aged ≥65 years from an urban community who were enrolled in participating primary care practices, and who were at high risk of heavily using health services during the following year.	To first improve patients’ quality of care and physicians’ satisfaction with the quality of care, which would later improve patients’ quality of life and efficiency in the use of resources for older adults with multimorbidity.	RN collaborated with the GP and primary care office staff, and coordinated the activities of all care providers involved.	Program included a CGA of the medical, functional, cognitive, affective, psychosocial, nutritional, and environmental status of older people.	Older people were involved in prioritizing care needs, and care plans were adjusted according to their priorities and preferences.
United States	Restorative Home Care [48, 49]	Older people aged ≥65 years who were cognitively unimpaired and at risk for functional decline after acute illness or hospitalization, but who had the potential for maintaining or improving their functioning.	Improve participants’ health outcomes, functional status and service use; and increase their likelihood of remaining at home.	Home care agency reorganized staff into multidisciplinary teams consisting of an RN, OT, PT and HHAs.	Reorientation of home care teams’ focus from primarily treating diseases and “taking care of” patients to that of working together to maximize functioning and comfort.	Goals and the process of reaching them was established based on input from and agreement between the patient, the family and home care staff.
The Netherlands	Carewell [50–52]	Frail older people ≥70 years from an urban community who were registered with participating general practices.	Improve quality of life and reduce functional decline, institutionalization, and hospitalization among frail older people living in the community.	Multidisciplinary team consisted of a GP, CCRN, gerontological social worker, elderly care physician, and a pharmacist. CCRN or social worker functioned as case managers.	Program targeted participants’ goals and needs on the domains of health and wellbeing	Case manager supported participants with goal setting and self-management by means of home-visits and telephone contacts.
The Netherlands	Embrace [53–55]	People aged ≥75 years from a semi-rural community who were registered with participating general practices.	Support older adults to age in place and improve health outcomes. Also improve quality of care and reduce service use and costs.	Multidisciplinary team consisted of GP, CCRN, social worker and elderly care physician. For complex and frail participants, the nurse or social worker acted as case manager.	Program took into account all aspects of participants’ functioning and disability, along with social and environmental aspects of their lives.	Older people were involved in identifying and prioritizing care needs, and the case manager helped navigate them through the processes of organizing the appropriate care and support.
The Netherlands	FIT [56, 57]	People aged ≥70 years at increased risk of functional decline who were registered with participating general practices.	Prevent or delay functional decline and disability.	Intervention was delivered by CCRN and GP. CCRN collaborated with other professionals when necessary.	Program included a CGA that addressed the somatic, psychological, functional and social domains of participants’ lives.	Older people were involved in prioritizing care needs and developing their care plans.
The Netherlands	U-Profit [58–60]	Potentially frail people aged ≥60 years who were enrolled in participating general practices.	Improve daily functioning of older people receiving primary care.	Program was delivered by an RN and GP. The RN collaborated with other professionals when necessary.	Program addressed biopsychosocial care needs.	During home visits, the RN focused on participants’ perceived care needs and preferences.
Sweden	Elderly Persons in the Risk Zone [61–65]	People aged ≥80 years from an urban community who were cognitively unimpaired and not dependent on help with ADLs.	Delay the progression of frailty in older adults, preserve their health and quality of life, and minimize their need for health care.	Nurse, PT, OT and social worker collaborated to perform preventive home visits or facilitate group meetings for older people.	Preventive home visits and group meetings focused on the physical, psychological, social and environmental domains of participants’ lives.	Participants’ experiences formed the basis of the group meetings; they were the experts, whereas the professionals functioned as enablers.
Sweden	Home-based Case Management [66–71]	Older people ≥65 years living in one municipality containing both urban and rural areas, and who were cognitively unimpaired and had at least two ADL dependencies.	Decrease participants’ healthcare use and improve their life satisfaction and other outcomes.	RN delivered case management in collaboration with PT, municipal health and social care services, GPs and the university hospital.	Comprehensive assessment addressed multiple key domains such as function, health, social support, and services.	Older people were consulted regarding the goals and needs that are important to them, and the care activities implemented for them.
Switzerland	SpitexPlus [72]	People aged ≥80 years from an urban community who were cognitively unimpaired.	Promote self-care ability and skills for a home-based population aged 80 years and older.	The program was delivered by advance practice nurses who were trained by geriatric specialists.	Program identified health problems as well as social problems.	Program targeted the problems older people chose to focus on.

Abbreviations: *ABLE* Advancing Better Living for Elders, *OT* Occupational Therapist, *PT* Physical Therapist, *CAPABLE* Community Aging in Place—Advancing Better Living for Elders, *RN* Registered Nurse, *(I) ADL* (Instrumental) Activities of Daily Living, *ED* Emergency Department, *GP* General Practitioner, *HHA* Home Health Aides, *CGA* Comprehensive Geriatric Assessment, *CCRN* Community Care Registered Nurse, *FIT* Functioning in Transition

**Table 2:** Safety risks addressed in integrated care programs for older people living at home

	Client functioning	Client behaviour	Social environment	Physical environment	Health and social care management
ABLE	Fall risk	Self-management		Obstacles and hazards in the home	
CAPABLE	Fall risk, functional impairments, depressive symptoms	Self-management		Obstacles and hazards in the home	Polypharmacy, patient-provider communication
Guided Care	Fall risk, functional impairments	Self-management, dietary intake, substance abuse	Caregiver burden, elder abuse		Care continuity and transitions, access to care
Restorative Home Care	Functional impairments	Self-management	Caregiver burden	Obstacles and hazards in the home	Polypharmacy, care continuity and transitions
Carewell	Fall risk, functional impairments, cognitive impairments, depressive symptoms	Dietary intake			Polypharmacy, care continuity and transitions, over- and under treatment
Embrace	Functional impairments	Self-management	Caregiver burden		Polypharmacy
FIT	Fall risk, functional impairments, cognitive impairments, depressive symptoms	Medication management, dietary intake, substance abuse	Social isolation, caregiver burden, elder abuse	Obstacles and hazards in the home	Polypharmacy, care continuity and transitions
U-Profit	Fall risk, functional impairments, cognitive impairments, depressive symptoms	Dietary intake	Social isolation, caregiver burden		Polypharmacy
Elderly Persons in the Risk Zone	Fall risk, functional impairments	Self-management, medication management, dietary intake	Social isolation	Obstacles and hazards in the home	
Home-Based Case Management	Fall risk, functional impairments	Medication management, dietary intake	Social isolation	Obstacles and hazards in the home	Polypharmacy, care continuity and transitions, access to care
SpitexPlus	Functional impairments, cognitive impairment	Dietary intake	Social isolation		Care continuity and transitions

Abbreviations: *ABLE* Advancing Better Living for Elders, *CAPABLE* Community Aging in Place—Advancing Better Living for Elders, *FIT* Functioning in Transition

Note: empty cells indicate that identified literature provided no indication of risks in these domains being addressed

**Table 3:** Activities, interventions and tools used in integrated care programs to address risks on different domains of safety

	ABLE	CAPABLE	Guided Care	Restorative Home Care	Carewell	Embrace	FIT	U-Profit	Elderly Persons in the Risk Zone	Home-Based Case Management	SpitexPlus
Activities targeting client functioning											
Assessment of risks and needs	X		X	X	X	X	X	X		X	X
Evidence based guidelines and protocols to address risks and needs			X	X	X	X	X	X			X
Proactive risk management	X		X	X		X				X	X
Activities targeting client behaviour											
Training in disease and symptom management			X			X					X
Training in medication management		X								X	
Training in functional performance	X	X		X						X	
Education about potential problems and risks	X			X		X			X	X	X
Counselling in how to cope with problems and risks		X		X	X				X		X
Activities targeting the social environment											
Education and counselling for caregivers			X	X							
Access to community resources			X						X	X	
Activities targeting the physical environment											
Aids and assistive devices	X			X						X	
Adjustments and repairs in the home	X	X		X						X	
Activities targeting health and social care management											
Medication reviews		X		X	X					X	
Coordination activities within care teams		X		X	X	X				X	
Case management during care transitions			X		X	X	X			X	
Training on specific aspects of safety			X	X	X	X	X	X		X	X

Abbreviations: *ABLE* Advancing Better Living for Elders, *CAPABLE* Community Aging in Place—Advancing Better Living for Elders, *FIT* Functioning in Transition

Note: empty cells indicate that this type of activity was not reported in the identified literature as being part of the integrated care program

With regard to the components of integrated care, *interdisciplinarity* of the programs varied. Programs included staff from two to five different health and/or social care professions. Nurses (*n* = 10) were the most commonly represented, followed by physicians (n = 6) and physical or occupational therapists (*n* = 5). Social workers were only included in three programs. As pertains to the principle of *comprehensiveness*, the included programs paid attention to participants’ needs across multiple domains of life. These included, amongst others, physical health and functioning (*n* = 11), social needs and well-being (*n* = 8), psychological and cognitive health (*n* = 5), and the physical environment (n = 5). All eleven programs observed the principle of *person-centeredness* by focusing on the problems, needs, or goals identified and/or prioritised by participants. Some programs also involved participants in developing or providing input on their care plans, and on the intervention-related activities (n = 5); and promoted participants’ involvement in managing their care (*n* = 1) and organising appropriate care and support for themselves (n = 1).

### Domains of safety risks addressed in included programs

Table 2 provides an overview of the risks that were addressed on the five different domains of safety risks, namely: client functioning, client behaviour, social environment, physical environment, and health and social care management. We considered a risk to have been addressed in some way or form if it was described in the study. Generally, the safety risks identified in this review were interpreted as safety risks by the reviewers—the risks were not labelled as safety risks in the included studies themselves. Even though the findings show that a variety of safety risks were addressed, overall, we found no clear patterns related to the stated program goals and the safety domains or types of risks they addressed.

We found that all eleven programs addressed at least three domains of safety risks; nine programs addressed four out of five domains, and three addressed all five domains of safety risks. All programs addressed risks related to *client functioning* and *client behaviour*, whereas risks related to people’s *social environment* (*n* = 8) and *physical environment* (*n* = 6) were addressed in about two-thirds of the programs. Finally, nine programs addressed risks related to *health and social care management*. We found the programs to be rather uniform in the types of risks they addressed within each domain. For example, the risks related to client functioning that were addressed generally pertained to either functional impairments, fall risk or both. Cognitive impairments and depressive symptoms were addressed less often. For client behaviour, there was somewhat more variation across the programs in the types of risks addressed. Whereas risks related to self-management and dietary intake were addressed in more than half of the programs, risks related to substance abuse and medication management were addressed in only two and three programs, respectively. Risks related to people’s social environment mainly included social isolation or caregiver burden, and risks related to peoples’ physical environment that were addressed always consisted of obstacles and hazards in the home. Care continuity and transitions, and polypharmacy, were most often addressed in the health and social care management domain, whereas safety aspects such as access to care, patient-provider communication, and over- and under treatment were only targeted sporadically.

### Activities and interventions used to address safety risks

Table 3 provides an overview of the activities and interventions used within the eleven programs to address safety risks on different domains. We considered an activity to be a part of the given integrated care program if it was described as so in the study. We note again that safety-related activities and interventions were mainly identified and interpreted as being safety-related activities and interventions by the reviewers because the programs themselves did not label the activities as safety-related. Activities targeting risks related to *client functioning* were quite uniform across the programs. They included comprehensive assessments of participants’ health risks and needs (*n* = 9), evidence-based guidelines and protocols for planning for care (*n* = 7), and proactive monitoring of participants’ functioning on the identified risks and needs (*n* = 6). Although all programs addressed risks related to client functioning (see also Table 2), for the programs Community Aging in Place—Advancing Better Living for Elders (CAPABLE) and Elderly Persons in the Risk Zone, no activities targeting these risks were described. Similar findings were observed regarding risks related to *client behaviour*. While all programs addressed risks in this domain, the programs Functioning in Transition (FIT) and U-Profit did not describe any associated activities or interventions. Overall, activities and interventions targeting risks related to client behaviour showed more variation across the programs compared to those targeting risks related to client functioning. Approximately half of the programs (*n* = 6) included written or verbal education for participants about potential problems and risks, whereas five programs provided participants with counselling on how to cope with behaviour problems and risks. In three cases, counselling was provided by registered nurses who acted as case managers. Furthermore, seven programs provided participants with various forms of training, such as in disease and symptom management (*n* = 3), functional performance (*n* = 4) and medication management (*n* = 2).

Activities targeting risks related to participants’ *social environment* were less frequently reported across the eleven programs. Of the eight programs that addressed such risks, only four described the associated activities. The programs Guided Care, Elderly Persons in the Risk Zone, and Home-Based Case Management addressed risks such as social isolation and elder abuse by helping people get access to community resources; and Restorative Home Care and Guided Care addressed caregiver burden by training and counselling caregivers. As for risks related to participants’ *physical environment*, four programs described activities that included the installation of adaptive equipment and/or adjustments and repairs in the home. In the programs Advancing Better Living for Elders (ABLE) and CAPABLE, a handyman was part of the comprehensive intervention; whereas in the programs Home-Based Case Management and Restorative Home Care the home safety activities (e.g., adjustments, repairs) were outsourced. Finally, activities aimed at risks related to *health and social care management* included, for example, taking action to improve coordination between different professionals. Programs did this by providing case management across care transitions (*n* = 5) or by implementing shared records or multidisciplinary team meetings (n = 5). Additionally, most programs (*n* = 8) trained the professionals involved. Training targeted, for example, how to deal with polypharmacy, provide counselling, and comprehensively assess risks and needs. Even so, only four out of seven programs that addressed polypharmacy included an activity related to medication review.

## Discussion

This scoping review aimed to provide insight into how integrated care programs for older people living at home address a broad range of safety risks. After examining the programs according to a predefined conceptual framework, our findings suggest that all included programs addressed risks related to client functioning and behaviour, and several programs addressed risks related to health and social care management. Fewer programs addressed risks related to people’s social and physical environments as evidenced by program activities and interventions. However, as the number of people receiving care and support at home keeps rising, risks related to, for example, people’s socio-economic conditions, their home environments, and increasing caregiver responsibilities, are becoming more prominent [24]. Addressing such risks is especially important since older people themselves also express that such concerns influence their ability to age in place [20, 25].

Our findings show that integrated care programs vary in the extent to which they addressed safety risks in a multidimensional way. Programs were quite heterogeneous in terms of their activities and interventions for addressing safety risks, but overall, the activities and interventions fell into two broad categories. Some activities and interventions were relevant for preventing harm that may arise from health care interventions, for example, through the organisation of medication reviews or training for professionals. Other activities were pertinent for improving safety by preventing (unnecessary) health decline and supporting people to manage risks in their daily lives. While both types of interventions are necessary in order to address safety, we found that few programs were as comprehensive as to include activities and interventions targeted at *all* domains of safety risks. Incorporating additional interventions targeting specific risks such as social isolation [73], caregiver burden [74] or environmental hazards [75] might improve the programs’ ability to support older people to live safely at home.

### Methodological considerations

Scoping reviews differ from systematic reviews in that they are used to answer questions related to the identification and mapping of certain characteristics or concepts, rather than answering a strictly defined clinical question. Nevertheless, the systematic approach to executing the research and reporting the study results is similar between the two types of reviews [33]. However, this review has some methodological limitations that should be considered when interpreting the findings. Although we performed a broad literature search to identify as many relevant papers as possible, our search strategy was constrained to a specific timeframe and three languages. Consequently, we may have missed studies published before 2007 or in languages other than English, Dutch, and Norwegian. Nevertheless, considering that research on both integrated care programs for older people [76, 77] and safety in primary and community care [23, 24, 32] became increasingly prevalent from 2007 onwards, we expected to find most of the relevant papers within this review’s timeframe.

We acknowledge the limitation that the selection process depended on relevant information being reported in the identified publications. Since we searched for content rather than outcomes of integrated care programs, we purposefully included program protocols and descriptions, as well as process evaluations, in addition to outcome evaluations. Still, several integrated care programs were excluded because information on one or more of the selection criteria was not available in the papers written about the programs. This was especially the case for the criteria of person-centeredness and safety. Given that space in scientific publications is often limited, we cannot rule out the possibility that some excluded programs did in fact address but did not report the inclusion criteria in question. Furthermore, both ‘safety’ and ‘integrated care’ are elastic concepts that can be conceptualised and defined in different ways. Variations in definitions may result in slightly different subsets of the literature being included for review.

When interpreting the findings of this review, it is important to realise that the programs we identified did not necessarily aim to address older people’s safety at home. We used a predefined conceptual framework, based on Lau et al.’s framework for health-related safety and other literature, to review how existing integrated care programs addressed the safety of older people living at home. This approach enabled us to look at existing data from a new perspective. Nevertheless, a limitation of our framework is that it only encompasses an objective approach to safety. It brings together all the different risks and problems that could potentially undermine people’s safety at home. However, older people’s feelings about their safety are not included in the framework. There are possibly significant discrepancies between older people’s objective and subjective safety [20, 29, 78]. Based on this review, it is not possible to indicate the extent to which integrated care programs address the subjective safety of older people living at home.

### Implications for research and practice

The findings of this review suggest that addressing safety is currently an implicit aspect rather than explicit objective of integrated care programs for older people living at home. This is not necessarily a negative observation, considering that many professional disciplines have their own safety priorities and protocols that they observe. Nevertheless, in light of our reflections on the results of this scoping review, more explicit consideration of safety in the context of integrated care programs for older people living at home could have several advantages. For example, explicit consideration of the multidimensional nature of safety risks might reduce unintentional oversight among professionals delivering primary and community care, and as such increase the chances that risks are comprehensively addressed. Additionally, explicitly addressing safety risks in an interdisciplinary and multidimensional way could enhance professionals’ understanding of how multiple risks are interrelated and accumulate to undermine older people’s safety. Such work would require that an interdisciplinary team of professionals collaborate and look within and beyond their individual areas of expertise to identify and address safety needs and priorities. Multidimensional risk assessments and innovative ways of involving social care and tapping into community resources would broaden the capacity of integrated care programs to promote older people’s safety in an interdisciplinary and comprehensive way.

However, it is important to acknowledge that safety is not the only aspect to consider in primary and community care for older people. While safety is an important precondition for good quality care and support [28], a strong focus on safety may have adverse effects on older people’s quality of life [79]. Safety should not be considered in isolation from other things that older people value, such as autonomy and the ability to live their lives as they wish [20, 79]. As such, many safety risks cannot be addressed independently of older people’s perspectives [80], which implies a need for open communication and trust between professionals and older people. Professionals should strive for a person-centred way of working, creating an environment where both they and the older people they serve can successfully collaborate to identify safety needs and solutions that take into consideration older people’s priorities and what is appropriate and sustainable for them [28].

This scoping review aimed to provide insight into how integrated care programs address safety risks for older people living at home. Assessing the programs’ effects on safety was not within the scope of this review. Even though some studies did include assessments of program effects on specific risks such as physical functioning or fall risk, comprehensive evaluations of programs’ impact on older people’s safety, from a multidimensional perspective, are necessary to gain insight into the programs’ ability to support older people to live independently at home. The complexity of integrated care programs do pose challenges for such evaluations, as programs often vary in terms of the type of program, the frequency and duration of activities implemented, and the dynamic contexts in which they are implemented. Therefore, in addition to the traditional intervention studies, future research might also consider alternative methods for evaluation [28] such as case study designs [81] or realist evaluation approaches [82]. Such designs, which often employ mixed methods, would facilitate investigation of complex phenomena as they occur in everyday contexts, and bring researchers closer to answering questions as to whether integrated care programs improve older people’s safety, and how.

## Conclusion

This review showed that integrated care programs included in the study addressed a broad range of safety risks for older people. Most attention was paid to risks related to older people’s functioning, behaviour, and health and social care management. However, risks related to older people’s social and physical environments are becoming increasingly important and need more attention. Prioritising a multidimensional approach to safety in integrated care programs could add value to the ability of primary and community care providers to support older people to live safely at home. Integrated care provides a platform and tools that could be harnessed further to address safety for older people living at home—in a manner that balances safety with other values important to older people, and that compliments and fills the gaps in discipline-specific safety measures and approaches.

## Supplementary information

**Additional file 1.** Detailed search terms and steps. This file presents the search terms used and steps taken during the literature search.

## Data Availability

Not applicable.

## Abbreviations

ABLE: Advancing Better Living for Elders
CAPABLE: Community Aging in Place—Advancing Better Living for Elders
FIT: Functioning in Transition
PRISMA: Preferred Reporting Items for Systematic Reviews and Meta-Analyses
