# Supplementary material for: Addressing safety risks in integrated care programs for older people living at home: a scoping review
Source: BMC Geriatr. 2020 Feb 28;20:81. doi: 10.1186/s12877-020-1482-7 (PMC7048120; doi:10.1186/s12877-020-1482-7)
Supplement: Supplementary file 1 — Additional file 1. Detailed search terms and steps. This file presents the search terms used and steps taken during the literature search. [file 12877_2020_1482_MOESM1_ESM.pdf]

Addressing safety risks in integrated care programs for older people living at home: a scoping review
Manon Lette, Eliva A. Ambugo, Terje P. Hagen, Giel Nijpels, Caroline A. Baan, Simone R. de Bruin

Additional file 1 – Detailed search terms and steps

Table A1. Embase search strategy[1]

Step	Search terms for search conducted on 8 March 2018[2]
#1	'old people':ti OR 'elder people':ti OR 'older people':ti OR 'older adults':ti OR 'retired adults':ti OR 'older persons':ti OR 'older patients':ti OR 'retired persons':ti OR 'retired older':ti OR 'retired people':ti OR 'old age':ti OR oldest:ti OR aged:ti OR aging:ti OR ageing:ti OR elderly:ti OR elders:ti OR seniors:ti OR 'senior citizens':ti OR 'later life':ti OR 'over 65':ti OR '65 and over':ti OR 'aged 65':ti OR 'older than 65':ti OR 'young-old':ti OR 'retir* baby boomers':ti OR 'ag*ing baby boomers':ti OR 'older baby boomers':ti OR geriatric*:ti OR frail*:ti
#2	'frail elderly'/de OR 'very elderly'/de OR 'aged'/de OR 'aging'/de OR 'geriatric care'/de OR 'geriatric nursing'/de OR 'elderly care'/de OR 'geriatrics'/de
#3	#1 or #2
#4	((living NEAR/5 community):ti,ab) OR ((live NEAR/5 community):ti,ab) OR 'community dwelling':ti,ab OR 'home dwelling':ti,ab OR ((living NEAR/5 home):ti,ab) OR ((live NEAR/5 home):ti,ab) OR 'assisted living':ti,ab OR 'sheltered housing':ti,ab
#5	'community living'/de OR 'independent living'/de OR 'assisted living facility'/de OR 'home care'/de OR 'home mental health care'/de OR 'home monitoring'/de OR 'home visit'/de OR 'visiting nursing service'/de OR 'home rehabilitation'/de
#6	#3 AND (#4 OR #5)
#7	((comprehensive NEAR/5 care):ti,ab) OR ((integrated NEAR/5 care):ti,ab) OR 'care coordination':ti,ab OR 'coordinated care':ti,ab OR 'multifactoral care':ti,ab OR 'multifactoral intervention*':ti,ab OR 'case management':ti,ab OR 'comprehensive health care':ti,ab OR ((continuity NEAR/5 'care'):ti,ab) OR 'critical pathways':ti,ab OR 'care pathways':ti,ab OR 'patient care management':ti,ab OR 'patient care planning':ti,ab OR 'patient care team':ti,ab OR 'managed care':ti,ab OR 'guided care':ti,ab OR 'integrated care':ti,ab OR 'long term care':ti,ab OR 'chronic care':ti,ab OR 'patient centred care':ti,ab OR 'patient centered care':ti,ab OR 'patient oriented care':ti,ab OR 'patient centredness':ti,ab OR 'person centred care':ti,ab OR 'person centered care':ti,ab OR 'person oriented care':ti,ab OR 'person centredness':ti,ab OR 'shared care':ti,ab OR 'transmural care':ti,ab OR 'multidisciplinary program*':ti,ab OR 'interdisciplinary program*':ti,ab OR 'proactive care':ti,ab OR 'pro-active care':ti,ab
#8	'integrated health care system'/de OR 'integrated care'/de OR 'patient care planning'/de OR 'advance care planning'/de OR 'managed care'/de OR 'managed care organization'/de OR 'long term care'/de OR 'care coordination'/de OR 'case management'/de OR 'patient care'/mj
#9	#6 AND (#7 OR #8)
#10	'home care safety':ti,ab OR ((safety NEAR/4 'home care'):ti,ab) OR ((safety NEAR/4 home*):ti,ab) OR ((safety NEAR/4 environment):ti,ab) OR 'safe environment*':ti,ab OR 'safe home*' OR 'health-related safety':ti,ab
#11	'patient safety':ti,ab OR 'patient safety'/mj OR 'harm reduction'/de OR 'risk reduction'/mj OR 'risk management'/de OR 'total quality management'/mj OR 'quality improvement':ti,ab
#12	('patient safety'/de OR 'risk reduction'/de) AND ('intervention study'/de OR 'multidisciplinary intervention*':ti,ab,de OR 'multi-agency intervention*':ti,ab,de OR 'integrated interdisciplinary':ti,ab,de OR 'health program'/de OR 'program evaluation' OR 'quality

	adjusted life year'/de OR 'quality of life'/de OR 'conceptual framework'/de)
#13	(('medication error'/de OR 'polypharmacy'/de OR 'malnutrition'/de OR 'loneliness'/de OR 'social isolation'/exp OR 'emotional deprivation'/de OR 'deterioration'/de) AND 'mortality'/de OR 'death'/de OR 'accidental death'/de) AND 'prevention'/lnk
#14	(('medication error'/de OR 'polypharmacy'/de OR 'malnutrition'/de OR 'loneliness'/de OR 'social isolation'/exp OR 'emotional deprivation'/de OR 'deterioration'/de) AND 'mortality'/de OR 'death'/de OR 'accidental death'/de) AND ('intervention study'/de OR 'multidisciplinary intervention*':ti,ab,de OR 'multi-agency intervention*':ti,ab,de OR 'integrated interdisciplinary':ti,ab,de OR 'health program'/de OR 'program evaluation' OR 'quality adjusted life year'/de OR 'quality of life'/de OR 'conceptual framework'/de)
#15	((reduce* OR reducing OR reduction OR decreas* OR minimiz* OR prevention OR preventing OR prevent OR avoiding) NEAR/5 (preventable OR avoidable OR predictable OR unintended OR unnecesary OR 'adverse outcome*' OR 'adverse events' OR errors OR incidents OR mistakes OR 'health risks' OR harm OR hazards OR injury OR injuries OR problems OR decline OR deterioration OR hospitalization* OR 'hospital utilization' OR admissions OR institutionalization OR 'emergency department*')):ti,ab
#16	(((decline OR deterioration) NEAR/3 (health OR functioning)):ti,ab) OR ('detorioration' AND 'health status'/de)
#17	((reduce OR reducing OR reduction OR decreas* OR minimiz* OR prevention OR preventing OR prevent OR avoiding) NEAR/5 (overtreatment OR 'over-treatment' OR undertreatment OR 'under-treatment' OR polyphamacy OR polydrug* OR 'poly-drug*' OR errors OR mistakes OR incidents OR falls)):ti,ab
#18	((promot* OR maximiz*) NEAR/4 (recovery OR 'health maintenance' OR wellbeing OR 'well being' OR competence OR autonomy OR independence OR resilience OR resources OR 'active support' OR 'proactive support' OR 'proactive care' OR 'pro-active care' OR 'treatment-adherence' OR 'medication adherence')):ti
#19	('home visit'/de OR 'preventive home visit*':de) AND ('program evaluation' OR 'quality adjusted life year'/de OR 'quality of life'/de)
#20	safety:ti OR harm:ti OR errors:ti OR incidents:ti OR events:ti OR hazards:ti OR misktakes:ti OR risk*:ti
#21	#9 AND (#10 OR #11 OR #12 OR #13 OR #14 OR #15 OR #16 OR #17 OR #18 OR #19 OR #20)
#22	#21 AND [2007-2018]/py
#23	#22 AND (english:la OR dutch:la)

1. A similar search strategy was used for the search in Medline database.
2. This search was also performed including the Norwegian language, which resulted in zero additional hits.
